# A Review of Force Myography Research and Development

**DOI:** 10.3390/s19204557

**Published:** 2019-10-20

**Authors:** Zhen Gang Xiao, Carlo Menon

**Affiliations:** Menrva Research Group, Schools of Mechatronic Systems Engineering and Engineering Science, Simon Fraser University, Metro Vancouver, BC V3T 0A3, Canada; zgx@sfu.ca

**Keywords:** force myography, FMG, muscle, wearable, motions, human-computer interface, signal processing

## Abstract

Information about limb movements can be used for monitoring physical activities or for human-machine-interface applications. In recent years, a technique called Force Myography (FMG) has gained ever-increasing traction among researchers to extract such information. FMG uses force sensors to register the variation of muscle stiffness patterns around a limb during different movements. Using machine learning algorithms, researchers are able to predict many different limb activities. This review paper presents state-of-art research and development on FMG technology in the past 20 years. It summarizes the research progress in both the hardware design and the signal processing techniques. It also discusses the challenges that need to be solved before FMG can be used in an everyday scenario. This paper aims to provide new insight into FMG technology and contribute to its advancement.

## 1. Introduction

Information about the position or movement of a user’s limb can be used to monitor physical activities or for human-machine-interface applications. Such information can be extracted externally from the user using camera technology or through a wearable approach using sensors worn by the user. Compared to the camera approach, the wearable approach is not constrained to a fixed environment and it offers greater freedom to the user in terms of mobility. Among many wearable approaches, the one that relies on force myography (FMG) has gained ever-increasing traction among researchers over the past 20 years.

FMG is a non-invasive technique to decipher the position or movement of a limb based on changes in the stiffness of the corresponding musculotendinous complex (MC) against a default state. The changes in stiffness of the targeted MC is often monitored by placing a force transducer on the targeted location with a preload force. This preload force corresponds to the default state, which is usually chosen as a state where the limb is in a relaxed position. However, depending on the application, the default state can be arbitrarily chosen. The basic principle of FMG is similar to the palpation technique that a clinician uses for identifying different muscles of a limb. For example, while we are squeezing one of our hands, we can feel some of the extensor muscles become stiffer by placing fingers on the bulk of the forearm. For FMG, we replace the fingers with force transducers. The stiffer the MC is, the higher the pressure the transducers will detect.

The term force myography, a.k.a. FMG, was first introduced in the work of Wininger et al. [1], but such a technique has been exploited as early as the 1950s [2]. Specifically, a device named “French Electric Hand” could be controlled by a pneumatic force transducer located within a transcranial amputee socket. A user could control the opening or closing of the gripper by contracting the residual limb muscles against the transducer. However, such an approach did not gain momentum due to the limitation of the sensor and computer technologies in those days.

Fast forward to the late 1990s, researchers once again reignited the interest of using such an approach for controlling prosthetic devices. For example, Abboudi et al. developed a soft socket that was embedded with three pneumatic pressure transducers for controlling a multi-finger prosthesis [3]. The input signals from the transducers were mapped to different commands based on signal energy. Instead of just opening and closing a gripper, this multi-sensor setup demonstrated the feasibility of controlling complex hand prosthesis through the FMG approach. Subsequently, the same research group was able to increase the number of transducers within the socket to allow more sophisticated control of the prosthetic devices [4,5,6].

Other than using pneumatic pressure sensors to detect the changes in muscle stiffness, researchers also proposed to use the resistive polymer thick film (RPTF) sensor as the main sensing element due to its miniature size and high affordability. For instance, in 2006, Lukowicz’s group demonstrated the potential use of the RPTF sensors to identify different limb movements, including both upper and lower limb actions [7,8]. The same group also demonstrated the potential use of such a technique to identify different activities of daily living [9]. Many of the early works between the late 1990s and 2000s focused on exploring the potential use of FMG by examining the signal morphology in a few limited scenarios. These works opened many untapped research opportunities in the fields of human-machine-interface and activity monitoring applications.

**Figure 1:**
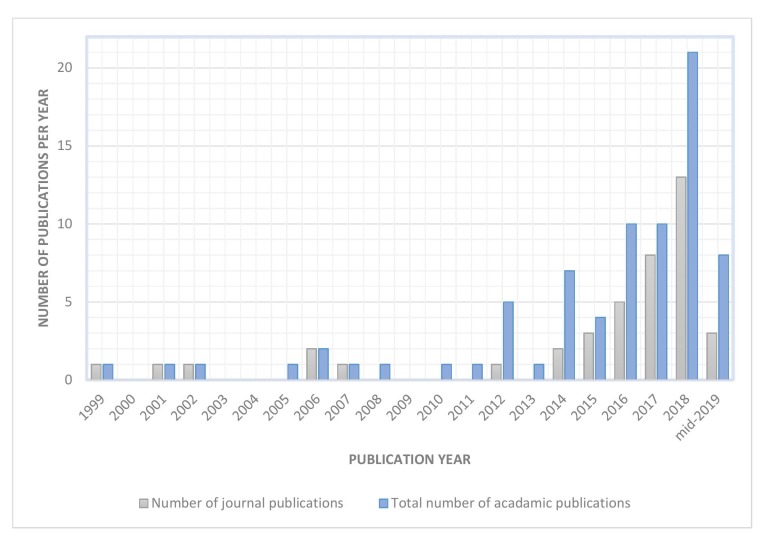
Number of FMG related publications from 1999 to mid-2019 (*n* = 76). The grey bar shows the number of journal publications and the blue bar shows the total number of FMG related publications which includes the journal, conference, and workshop proceeding articles.

Starting in the late 2010s, there was an increasing interest in the development of FMG technology, as shown by the number of academic publications depicted in Figure 1. A total of 76 papers were found between 1999 and mid-2019 (the list shown in Table A1 of the Appendix A).

This collection of FMG-related publications were obtained from the authors’ literature database and by searching the keywords “FMG”, “force myography”, “peripheral machine interface”, “muscle pressure”, and “force sensor” in Google Scholar. Among this collection, two papers are review articles that discuss topics related to the use of FMG for prosthesis control [10] and sensor fusion in hand rehabilitation [11]. it is important to note that FMG is only a popular term that describes such an approach and other researchers have used names such as residual kinetic image [4], muscle pressure map [12], pressure distribution map [13], tactile myography [14], etc., to describe the same technique. To avoid confusion for the readers, the term FMG is used throughout this paper to describe the various techniques that use force sensors to detect the changes in stiffness of a musculotendinous complex (MC) against a default state. It is also important to distinguish between the FMG approach and an approach that detects the change in forearm shape for gesture prediction. FMG uses sensors that detect the change in normal force due the change in stiffness of the MC, while the other approaches use camera [15], stretch sensors [16], strain gauges [17], capacitive sensors [18], or other types of transducers to detect the cross-sectional or overall displacement of the muscles during different limb actions [19]. The change in arm shape is also a result of the change in different muscles during contraction, but is not the same as changes in muscle stiffness. In an isometric scenario, the change in muscle stiffness does not necessarily translates to a change in the shape of the arm. Some researchers see the pattern in the change in arm shape as the same as the one for the FMG approach, but due to their fundamental difference, the two approaches should be analyzed separately. In this paper, we focus on the works that use force transducers as the main FMG sensing elements, not including the ones that only measure the deformation of the arm.

The objective of this review paper is to present the state-of-art research and development on FMG technology in the past 20 years. In the subsequent sections, we present the FMG signal acquisition and processing methods, followed by a discussion on the challenges that exist in FMG research, and its future direction.

## 2. FMG Signal Acquisition

**Figure 2:**
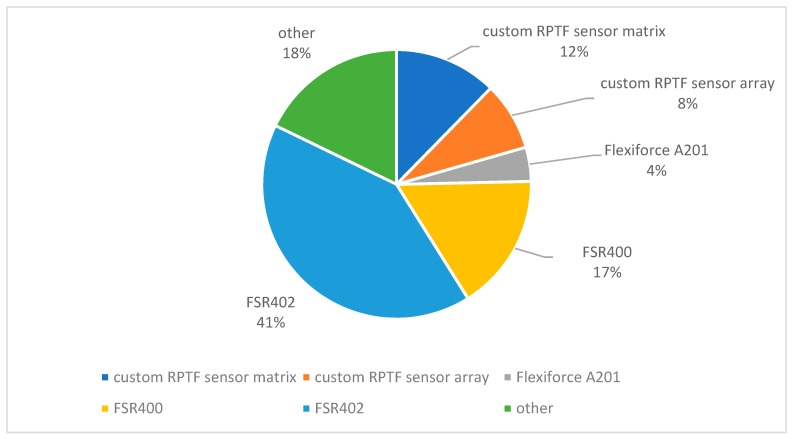
Percentage of the different FMG sensor types found in the literature (*n* = 73).

One key aspect of FMG research is how to convert the variation in the stiffness of MC to digital data for processing. To do that, we first need to select or develop force transducers to register the signals in their analog form, then convert these signals into digital data. The percentages of the different types of FMG sensors found in the literature are presented in Figure 2. The majority of the FMG sensors are based on resistive polymer-thick-film (RPTF) technology, which includes force sensing resistors (FSR), Flexiforce^®^ sensors, and other customized RPTF sensor arrays and matrixes. The rest include pneumatic-, piezoelectric-, capacitive-optical fiber-, and textile-based sensors.

In this section, we first focus on the different configurations of the RPFT sensors and the signal acquisition methods. We then review the different sensor placements for various applications. We subsequently present an example of FMG signals and compare the differences among the FMG signals and the signals of two well-established techniques that detect muscle activities, i.e., surface electromyography (sEMG) and mechanomyography (MMG). Finally, we discuss other types of novel FMG sensors.

### 2.1. Resistive Polymer Thick Film Sensor (RPTF) for FMG Application

#### 2.1.1. Element-Wise RPTF Sensor

**Figure 3:**
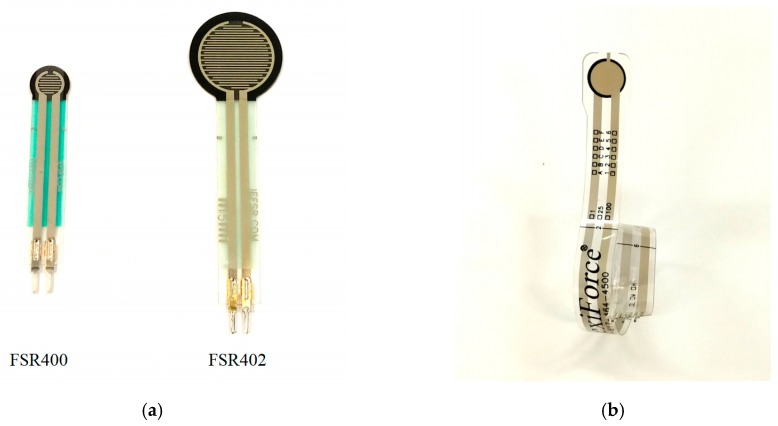
An example of the elementwise polymer thick film sensors (**a**) FSR sensors; (**b**) Flexiforce^®^ sensor.

FSR and Flexiforce^®^ sensors, as seen in Figure 3, are the most popular single element RPTF sensors used in FMG applications. In total, they account for 55% of the sensors found in FMG publications. They are both based on resistive polymer thick film technology, but with different configurations. The FSR sensor uses a shunt mode configuration in which two interdigitating electrodes are placed on top of a semi-conductive polymer layer. When a force is applied to the electrodes and the semi-conductive layer, the overall output resistance decreases, the thick film device acting as a force sensor. The Flexiforce^®^ sensor utilizes a thru mode configuration in which the semi-conductive layer is sandwiched between two flat electrodes. Similar to the FSR sensor, the resistance changes based on the total pressure applied to the sensing region on the electrodes.

A specification comparison chart between the two RPTF sensors primarily used in FMG research in the last 20 years is shown in Table 1. This information is based on the datasheets provided by Interlink Technology [20] and Tekscan [21]. There is no study that compares the actual performance of the two sensors for FMG applications, but based on the provided information, the FSR sensor is better on force repeatability and response time, while the Flexiforce^®^ sensor has a better hysteresis response. However, their test conditions are different, therefore, this information is for reference only.

**Figure 4:**
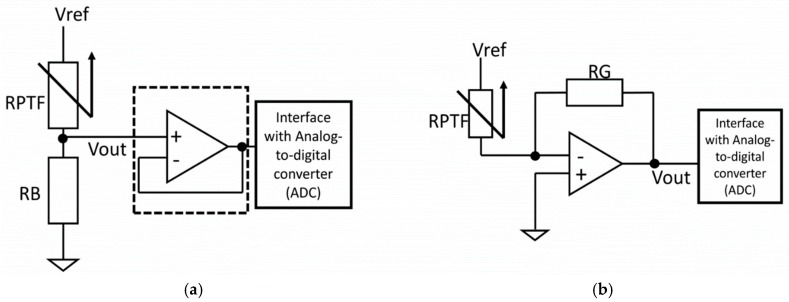
Circuit for extracting FMG signal. (**a**) Voltage divider setup (**b**) Current to voltage converter setup.

The most commonly used circuitry to extract the signal of the RPTF sensor is the voltage divider because of its simplicity. As shown in Figure 4a, the voltage divider setup only requires one bias resistor (RB) to condition the output voltage of the RPTF sensor. The bias resistor can be “pull-down” to the ground as shown in Figure 4a or “pull-up” to the Vref with one sensor terminal connecting to the ground. The output of the voltage divider can then be buffered using an op-amp with a unity gain configuration, before being digitized with a signal acquisition device such as a microcontroller with an analog-to-digital converter interface. However, the main drawback of using such circuitry is the non-linear response of the voltage output, which adds an unknown layer between the muscle stiffness and the sensor reading. This non-linearity also complicates the sensor drift characteristic as pointed out by Esposite et al. [22]. To reduce the non-linearity of the output response of the sensor, the manufacturer of the sensor has suggested using a current-to-voltage setup, as shown in Figure 4b. This setup has been adapted in some FMG papers, with slight variations [9,22,23,24]. In such a setup, an op-amp and a resistor (RG) for controlling the output gain have to be used. The voltage across the sensor is constant and the output voltage is proportional to the current passing through the sensor. This setup produces a more linear response across the full force range of the sensor. However, such circuit is not strictly needed if the desired characteristic is predetermined based on the targeted application. For example, the force range for FMG is typically under 2 N for the FSR402, which has a sensing area of 1.8 cm^2^. By selecting a proper value of the bias resistor, e.g., <10 kOhm for the FSR402, a voltage output response that has strong linear characteristics can be obtained within the functional range [25]. In some applications such as gesture prediction, the linearity characteristic of the signal plays a less important role, as long as the signal patterns are different among gestures.

**Figure 5:**
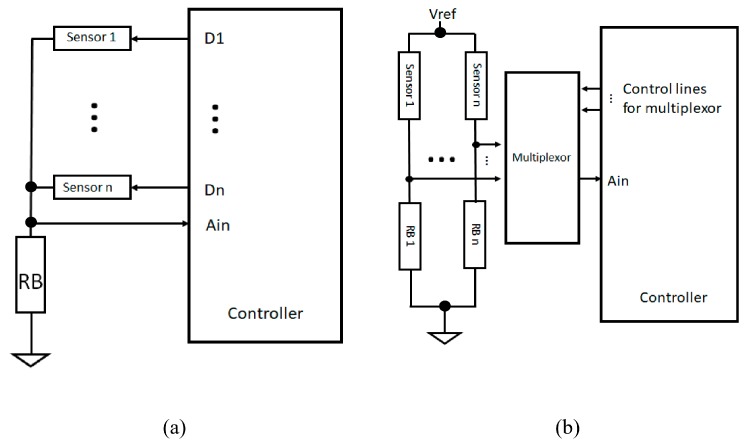
Examples of line scanning circuitries. (**a**) Configuration 1; (**b**) Configuration 2.

For most FMG applications, more than one sensor is needed to extract the stiffness pattern from different muscles. Researchers often insert the elementwise sensors into a strap or a socket to capture the pattern for gesture identification or to predict limb motion. The number of sensors can range from two to 32, or even more. Depending on the number of sensors, a customized signal acquisition device is needed. For a device with less than eight channels, researchers often directly replicate the voltage divider setup for each sensor and feed those signals directly to the ADC terminal of the signal acquisition device [26,27,28]. The reason for only eight channels is because many low-cost microcontrollers or signal acquisition devices are already equipped with eight independent analog input terminals, which simplifies the overall signal acquisition process. For more than eight inputs, the line scanning method is often adopted. Simply speaking, the line scanning method turns the sensors on-and-off sequentially and only allows the signal from one sensor at a time to be fed to the input terminal. Using such a method, the maximum sampling rate is lower compared to the parallel input setup because of the delay due to sensor switching and the fact that only one sensor at a time can be read. Two examples of the line scanning circuitries are shown in Figure 5a,b. For the circuity in Figure 5a, one of the sensor terminals is connected to a common line, which is injunction with a bias resistor (RB) and the analog input terminal (Ain). The other terminal of each sensor is connected to a tri-state digital pin. At any time, only one sensor is in the “on” state by setting the corresponding digital pin “High” and the rest in a high-impedance state. The reason for setting the rest in a high-impedance state instead of the “Low” state is to reduce cross-talk among the sensors [29]. Figure 5b shows a more commonly seen line scanning circuitry, which uses a multiplexor to control which sensor output can be read through the analog input terminal [30]. This multiplexor approach can be used for the voltage divider circuitry as well as for the current-to-voltage converter setup.

#### 2.1.2. High-Density Polymer Thick Film Sensor Array and Matrix

**Figure 6:**
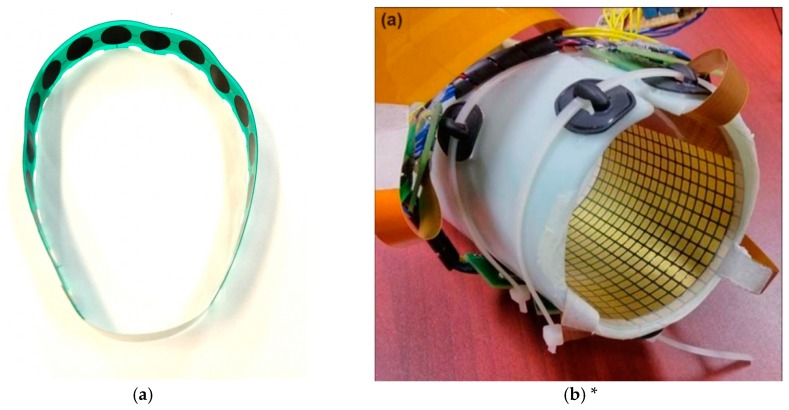
An example of a force sensing array and a matrix. (**a**) Force sensing array (**b**) Force sensing matrix. (* reproduced with permission from Radmand et al. [12]).

In recent years, researchers started to develop customized force-sensing arrays and matrices for FMG applications. Figure 6 shows two examples of such devices that are based on RPTF technology. The device in Figure 6a is a force sensing array developed by the MENRVA group at Simon Fraser University, which was utilized in the Cybathlon 2016 competition for controlling an upper limb prosthesis [31]. This array consists of 16 sensing elements, each element covers an area of 127 mm^2^ and has a gap of 6.6 mm between the adjacent elements. The device in Figure 6b is a high-density force-sensing matrix developed by the Institute for Biomedical Engineering at the University of New Brunswick, which debuted in 2014 [12].

This device consists of 14 × 9 sensing elements in a single matrix, each element covers an area of 10 mm × 10 mm. The recent work presented by Castellini et al., [14] also utilized the high-density force-sensing matrix approach to predict wrist and finger movement. Different from the one from the University of New Brunswick, the device presented in this work consisted of 10 separate rigid matrixes to cover the entire forearm circumference. Each matrix has 4 × 8 sensing elements, and each element covers an area of 5 mm × 5 mm. The working principles of the array and matrix are the same as their elementwise counterpart, but they allow researchers to capture more information relating to the activities of muscles with denser sensor configurations.

#### 2.1.3. Sampling Rate

The sampling rate of FMG varies among different publications. Depending on the objective of the work, the sampling rate ranges from 6 Hz to 1000 Hz. For static gesture prediction, 6 Hz may be sufficient, as we don’t expect the FMG signal to change drastically. However, for dynamic actions, low sampling rates may suffer from the aliasing effect and introduce error and loss of signal information. One of our recent publications suggests the minimum sample rate should be above 84 Hz based on empirical data [25]. In this study, two FMG straps with eight sensing elements were donned on the wrist and the forearm of 12 volunteers. The volunteers performed different hand actions as fast as possible while FMG was sampled at 1000 Hz. The results of this study showed that the signal extracted above 84 Hz had low discrepancy against the 1000 Hz signal.

#### 2.1.4. Sensor Placement and Applications

**Figure 7:**
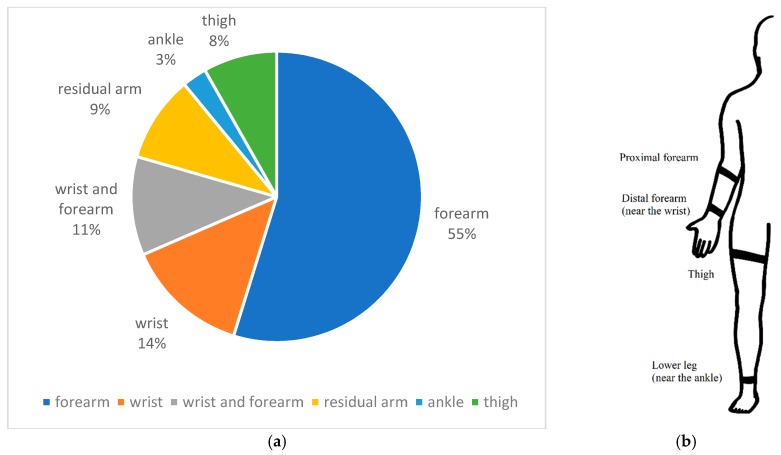
Information and depiction about the FMG sensor placement. (**a**) Percentage of sensor placement found in the literature (*n* = 73). (**b**) Depiction of FMG sensor placement.

As shown in Figure 7a, the majority of FMG related works are targeting the detection of upper limb movements from the musculotendinous complex (MC) of the forearm. For applications that target users with intact limbs, researchers often place an array of FMG sensors around the bulk of the forearm, near the wrist, or both as depicted in Figure 7b. The signals extracted from these two regions were shown to predict many hand gestures and continuous actions. For example, the work by Jiang et al. showed that 48 gestures could be predicted from either the bulk of the forearm or near the wrist, with an average of 90% cross-trial validations accuracy (*n* = 12) [32]. These 48 gestures included 16 grasp types, 16 sign language gestures, and 16 finger and hand movements. For continuous actions, Sadeghi et al. showed that the angle between index-and-thumb and the one between the middle finger-and-thumb could be accurately predicted using signals extracted near the wrist [33]. In this study, the authors also accounted for different wrist positions while predicting the angles. A correlation of determination (R^2^) of 0.871 was obtained for the index-and-thumb angle, and an R^2^ of 0.941 was obtained for the middle finger-and-thumb angle (*n* = 10). Also, when the term “FMG” was first introduced in 2008, it was already shown that continuous gripping force could be predicted using FSR sensors in a sleeve that covered the whole forearm [1]. Later in 2012, Yunger et al. used the same setup to predict grip force in a stroke rehab experiment aimed at improving fine motor function [34]. Not only was the gripping force predicted, but the pressing force for each finger could also be regressed with a high degree of accuracies, as demonstrated by Ravindra et al. [35]. With the ability to predict hand actions from the forearm muscles, it was shown that FMG could be used in monitoring functional activity of the upper limbs [28,29,36], controlling electromechanical devices [37,38], and even playing a virtual piano [39]. While most of the works related to human-machine-interface were aiming at applications that utilized an open-loop control strategy, researchers also started to investigate FMG in scenarios that involved dynamic interaction between the user’s hand and external robotic devices. For instance, an exploratory study by Sakr et al. showed that FMG could be used for admittance control applications [40]. In this study, a handler connected to a linear actuator could react to the applied force predicted from FMG sensors located on both the distal and proximal ends of the forearm. Although it was an exploratory investigation, it demonstrated the feasibility of using FMG for robotic interaction.

While the majority of FMG investigations use participants with intact limbs, a significant portion of these works actually targeted prosthesis control applications. In recent years, researchers successfully tested the FMG approach for predicting the intended actions of participants with trans-radial amputations [14,31,41,42,43,44]. For prosthesis control, the number of intended predicted actions is much less than the one for participants with intact limbs. In one of our works by Cho et al., we were able to predict up to 11 grips with various degrees of accuracies from four participants with trans-radial amputation. Within the 11 grips, five of them were primary grips used very often in activities of daily living. Including the default state, we were able to achieve above 70% averaged accuracies for the five primary grips. In order to increase the accuracy for practical use, we sub-divided the 11 grips based on the opposed thumb and non-opposed thumb modes and were able to achieve 89% accuracies for both settings. Using such a strategy, we were able to adopt the FMG approach to control a robotic prosthesis in a practical scenario, i.e., the 2016 Cybathlon competition [31].

For upper extremity action prediction (including participants with fully intact or amputated limbs), researchers often place the sensors around the proximal and mid-portion of the forearm, as the mass of the main extrinsic forearm muscles is most prominent in this region. However, FMG pattern on such a location is significantly influenced by the movements of the elbow [45]. In many scenarios, the movements of the elbow are not needed, and the FMG patterns associated with these movements become a confounding factor that needs to be filtered out. This problem is especially true in prosthesis applications since the control of the hand is the focus, not elbow action. Researchers have proposed different methods to filter such information. For gesture prediction, Radmand et al. suggested to collect hand gesture data with different upper arm and elbow locations, and then generate a prediction model that could account for the various scenarios [46]. On the other hand, Ferigo et al., used an additional sensor, i.e., an inertial measurement unit (IMU) to measure the elbow angle to compensate for the variation of the FMG signal and to improve grasping detection during dynamic arm movements [42]. There are very few works that discuss how the FMG pattern changes based on the elbow and shoulder positions, but it is an essential problem to be solved in order to use FMG in practical scenarios.

Researchers have shown that the FMG approach can be used to detect lower limb actions such as walking, as early as 2006. Specifically, Lukowicz et al. placed two pairs of FSR sensors at the front and the back of the thighs and studied the signal patterns during four types of locomotion which included normal walking, walking with an extra-long strike, as well as walking upstairs and downstairs [7]. The authors calculated the ratio of “back-to-front-leg” activity and the relative time delay of “back-leg” activity as features. Using the two features, it was shown that the four types of locomotion were separable. Despite the successful demonstration of using FMG for lower limb applications, there were little follow-up publications until 2011, in which Yungher et al. compared FMG signals and surface electromyography signals for gait cycle analysis [47]. The results showed that FMG signals were highly correlated to sEMG signals, and exhibited a more consistent pattern from stride to stride. Apart from walking, Belasis et al. used a similar FMG approach to study cycling activity and also compared the signal patterns against the one obtained through sEMG [48]. The study found that the two signals were highly correlated, but the FMG signal could reveal the overall level of fatigue better than the sEMG signal. Along with studying lower limb gross activity levels, researchers also investigated the possibility of using FMG to detect gait events by placing the sensors around the thigh or the ankle. For example, Godiyal et al. were able to predict different walking modes and gait events from the thigh [26,49]. The obtained accuracies for gait event detection were comparable to other technologies such as pressure insoles, inertial measurement units, and electromyography [49]. Besides the thigh, FMG signals extracted from the region near the ankle can also be used for gait event detection. Specifically, one of our works showed that seven ankle positions could be predicted with above 85% cross-trial accuracy [50]. We also investigated the factors influencing prediction accuracy. Among the many factors, the variation in stride length had the most influence on accuracy [51].

### 2.2. Example of FMG, MMG, and sEMG Signals from the Forearm for Squeezing Action

FMG is a relatively new technique to decipher activities related to muscle movements. Before that, researchers used techniques like surface electromyography (sEMG) and mechanomyography (MMG) for the same purpose. sEMG is a technique that registers electrical potentials resulting from muscle contraction caused by motor neuron firing [52], while MMG is a technique that captures vibrational characteristics during muscle contraction using an accelerometer or microphone [53,54]. We can consider that sEMG signals are the electrical manifestation of muscle movement, while MMG and FMG signals are their mechanical counterparts. MMG captures high-frequency information related to muscle vibration, while FMG captures movement information in the lower frequency range.

**Figure 8:**
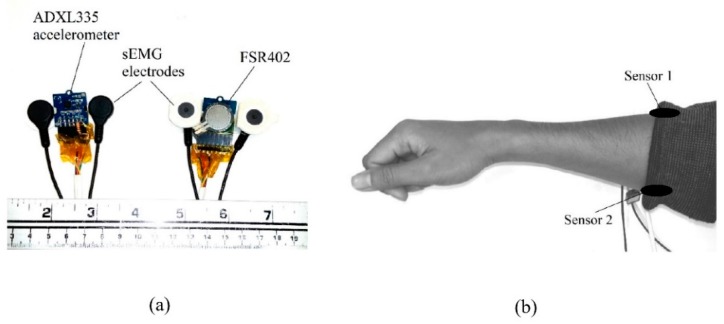
Setup to capture sample FMG, sEMG, and MMG signals. (**a**) FMG, sEMG, and MMG sensor (**b**) sensor placement.

In order to understand FMG signal morphology and how different is FMG from MMG and sEMG, we built two custom signal acquisition devices to capture those signals simultaneously in close proximity (see Figure 8a). Each device in the figure consists of one RPTF sensor (FSR402), one analog accelerometer (ADXL335), and a pair of sEMG electrodes from the Noraxon Myosystem 1400L sEMG amplifier. The FSR signal was extracted using a voltage divider circuitry with a 10 kOhm bias resistor. All signals were fed to a NI USB-6289 data acquisition device (DAQ) from National Instruments (city, state abbrev if USA, country) with a sampling rate of 2000 Hz. The two devices, i.e., Sensor 1 and Sensor 2, were placed on the extensor and flexor muscles of the forearm as shown in Figure 8b. The two sensors were secured to the forearm by a tight sleeve during data acquisition.

**Figure 9:**
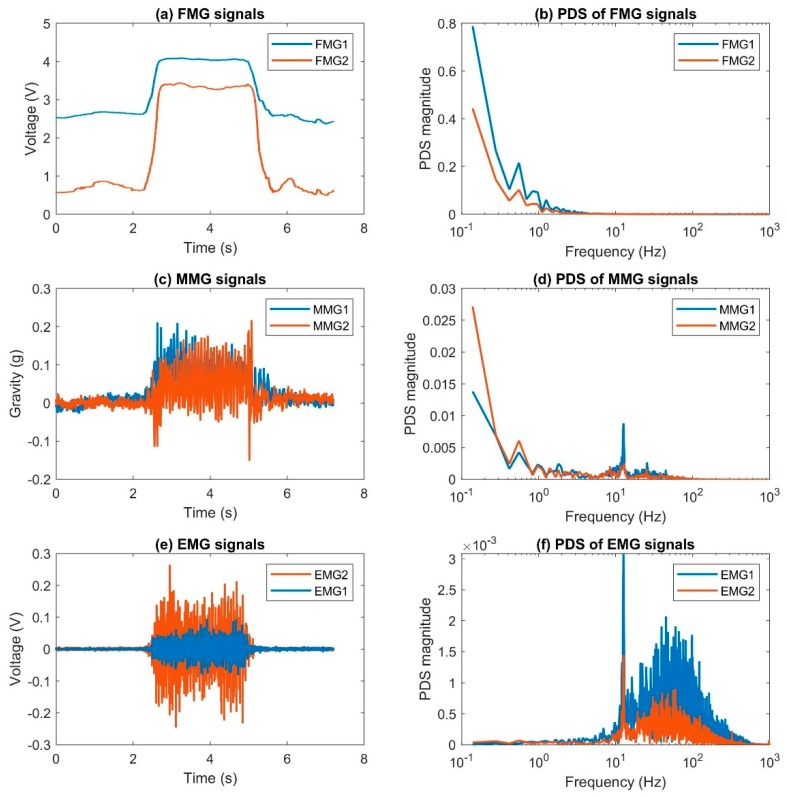
An example of FMG, MMG, and sEMG during a squeezing action (**a**,**c**,**e**). The blue lines show the signals that were captured from Sensor 1, the orange lines show the signals that were captured from Sensor 2. The x-axes of plot (**b**,**d**,**f**) are using the log scale.

An example of FMG, MMG, and sEMG signals from the forearm during an isometric squeezing action is shown in Figure 9. The blue lines show the signals captured from Sensor 1 while the orange lines show the signals captured from Sensor 2. The left column shows the captured signals in the time domain and the right shows the corresponding power density spectrum (PDS) using Fast Fourier Transform.

From the time domain plots, we can see that the squeezing action started after the 2-s mark and lasted about 3.5 s. During the squeezing action, the magnitude of the FMG signals remains constant, while the MMG and sEMG signals oscillate asymmetrically and symmetrically, respectively. In addition, we can see that the FMG signal energy mainly resides in the low-frequency range (<10 Hz), while that of the MMG has noticeable energy content before the 100 Hz mark. The majority of the sEMG signal energy is below 1000 Hz. This example illustrates the morphology of the three closely related signals. Since they reveal different aspects of muscle activity, we should not treat them as alternative techniques, but as complementary approaches. A comparison study of the different types of sensing techniques is valid only if it is for a specific scenario, not for general applications.

A recent study investigated the signals extracted from an FSR sensor at 10 kHz for FMG applications [22]. The authors captured the signal from the forearm with one single sensor and showed that it resembled the MMG signals after applying a high pass filter with a cutoff frequency of 2 Hz. They also compared the high-frequency FMG to sEMG and discussed the correlation. However, in order to show that the high-frequency signals can be used for FMG applications, we should capture signals from at least two different muscles to see if they are related to the targeted muscle activity and whether the captured patterns are separable among the different limb actions. Therefore, further investigation is needed to demonstrate the applicability of high-frequency FMG signals.

#### Other Force Sensors

Other than resistive based RPTF force sensors, researchers have developed different types of sensors for FMG applications. These sensors include the pneumatic-, resistive fabric-, piezoelectric-, capacitive-, and optical fiber-based force sensors. The soft socket presented in the work of Abboudi et al. was one of the early examples that used pneumatic principles to register the change in muscle stiffness [3]. This socket was made with silicon material with multiple air chambers connecting to electromechanical pressure transducers to obtain FMG patterns. The soft socket was custom-molded for each user, which allowed proper contact between the muscles and the chambers. The same research group later named such approach residual kinetic imaging (RKI) and studied the signal patterns obtained from individuals with amputated limbs, with the help of magnetic resonance imaging (MRI) technology [4]. However, the pressure within the chambers was difficult to maintain due potential leakage and changes in socket temperature. Also, the socket required sophisticated hardware to support the operation and therefore was cumbersome to use in practical everyday scenarios.

A highly flexible tactile sensor matrix for predicting gestures was developed by Rasouli et al., [55]. This sensor matrix was constructed with two pieces of stretchable fabric as the outer layers, two pieces of fabric with multiple conductive paths as the electrode pair layers, and one piece of piezoresistive fabric as the main sensing element. This matrix has a total of 16 × 8 sensing elements that fully cover an adult’s forearm. Its working principle was the same as the RPTF sensor with the thru mode configuration. The authors demonstrated that such a sensor could be used to predict hand opening and closing with different arm orientations.

Besides using resistive-based sensor technology, researchers have used and developed piezoelectric-based force sensors for FMG applications. Unlike the resistive-based sensor, which is a passive component, the piezoelectric transducer is able to generate electricity when a force-induced movement occurs within the piezoelectric elements that are sandwiched between two electrodes. The level of electricity generated is proportional to the speed of the deformation of the element, which is manifested as a voltage across the two electrodes. As a result, the piezoelectric sensor is only able to detect the movement; it cannot be used to predict static limb position without capturing the transitional pattern. Some examples of using piezoelectric sensors for FMG applications are summarized here. For instance, Li et al. used five flexible piezoelectric film sensors around the thigh to predict four leg movements, and they were able to achieve an accuracy as high as 92% across four participants [56]. Ha et al. used three piezoelectric sensors on the flexor carpi radialis, flexor carpi ulnaris, and brachioradialis muscles to predict four upper limb gestures [57]. They were able to achieve an average accuracy of about 80% across three participants. Booth et al. placed six sensors under the flexor tendons located near the wrist and they were able to predict five types of finger tapping with a 96% accuracy across 10 participants. Fang et al., fabricated their own custom piezoelectric force sensors for upper limb gesture prediction [58]. They characterized the sensor response and analyzed the signal morphology for seven upper limb gestures. An averaged accuracy of 96% across 8 participants was obtained for the gesture prediction.

Another novel force sensor used for FMG applications is the capacitive force sensor. This sensor measures the capacitance between two conductive plates when a normal force is applied to them. The capacitance is inversely proportional to the distance between the two plates, which is separated by a compressible layer made of a dielectric material. Based on such principle, Truong et al. developed a wrist band with 15 capacitive sensors for gesture control applications [59]. Their device was aimed at optimizing power consumption for the application. Using this device, they were able to predict 15 hand gestures with an averaged accuracy of 95% on 20 participants.

One more novel sensor used for FMG applications is based on the level of attenuation of light passing through an optic fiber. Specifically, Fujiwara et al. developed a force sensor that measures the change of light intensity from an LED light source within an optic fiber [60]. This sensor has an area of 60 × 10 mm^2^ with a total thickness of about 3 mm. It consists of two deforming plates, an optical fiber guider made with multiple graphite rods, and a 2 m long silica multimode optical fiber. The fiber is fitted inside a wavy space within the guider that is sandwiched between the two deforming plates. When a normal force is exerted on the deforming plate, the rods within the guider move closer and deform the optical fiber. The more deformation the fiber experiences, the harder it is for the light to pass through. By placing three of these sensors around the forearm, the authors were able to predict nine gestures with a 98% accuracy across six participants.

## 3. FMG Processing Methods

The goal for the majority of FMG applications is to predict limb actions or gestures from changes in the stiffness of the musculotendinous complex (MC). For a simple two state problem, such as distinguishing between a squeezing action versus a relaxed state while the arm is in a fixed position, a threshold value can be manually calibrated based on a single FMG reading to separate the two states. However, if we want to detect a squeezing action versus a relaxed state for various arm positions, a single threshold value will be difficult to obtain manually and may not be sufficient to obtain high prediction accuracy. Instead, most researchers rely on machine learning approaches to decipher the different limb actions from FMG signals. This approach requires collecting sample data and generating a decision model to link the collected FMG signal patterns to the targeted actions. Before a model is generated, researchers often condition the signal and extract useful information as the model input. Once the model is generated, it is used on the untrained data to predict the actions. Machine learning is a vast topic to discuss, in this paper, we only focus on the machine learning techniques that have been used for FMG applications. In this section, we will discuss the processing techniques used for pre-conditioning the input features and the model generation.

### 3.1. FMG Signal Conditioning and Feature Extraction

More than one FMG signals are usually recorded for limb action prediction applications. This set of signals forms the unique patterns that can be associated with different targeted actions. The raw FMG signals are usually represented in volt units or a digitized value that is associated with the resolution of the analog-to-digital converter. For ease of computing, the set of signals is scaled down to have a maximum range of 0 to 1 based on the largest reading recorded within the entire data set. This range is used because the FMG signal reading is always positive and it is not a bipolar signal that centers around zero. Such a step only changes the numeric representation of the signals and does not alter the signal pattern. Using these signals alone without additional signal conditioning or feature extraction steps, researchers were able to predict 48 static gestures with an averaged 90% cross-trial validations accuracy [51]. However, depending on the application, the prediction accuracy may have benefited from additional signal processing steps. These steps include signal filtering, normalization, and feature extraction.

FMG signals extracted from RPTF sensors usually have a high signal-to-noise ratio, as shown in Figure 9a. However, noise may be introduced to the electrical circuit due to instrument artifacts. Therefore, some researchers apply a low-pass filter with a cut-off frequency range from 4–20 Hz to further smooth out the signals [1,13].

The FMG signal pattern is highly dependent on the preloaded force, which is difficult to control during the donning of the sensors. In order to reduce this discrepancy, researchers usually normalize each signal by subtracting the signal from its mean value then dividing it by its standard deviation. Such a process is often referred to as auto-scaling. The auto-scaling step balances the significance of each input signal before the model generation process. However, it is important to ensure that each input signal has a non-zero standard deviation; otherwise, this process will fail as the centralized signal would be divided by zero. To avoid this problem, a channel selection step can be used to remove channels that do not capture meaningful information throughout the process.

Once the FMG signals are scaled or normalized, different signal features can be extracted. In general, there are two types of features, the instance feature, and window-based feature. The instance feature is extracted from a single instance in a set of multi-channel FMG signals. The mean, the standard deviation, the median, and the different percentiles of the set are some examples of instance features [61]. The window-based feature is extracted from the signals over a time window. Such a window consists of at least two data points and often more. There are many features that can be extracted from the time-based window. The mean magnitude, the average slope of a signal, the spectrum magnitude of a selected frequency band, and the coefficients of a polynomial approximation of a signal segment are some of the examples. Depending on the selected features, some of them can be computed from a single channel and some of them can be computed from the signal set. The value of a window-based feature depends on the selected window size; therefore, it is important to optimize the feature window for the targeted application [29]. Currently, only a limited number of publications extracted features from the FMG signals for targeted applications [14,29,45,51,58,61,62]. The optimal feature set is highly dependent on the application and it is difficult to identify a universal feature set for FMG signals. However, researchers can start with a set of features borrowed from other fields, such as the features used in sEMG, and then use optimization techniques to select useful features for the custom application. Researchers can also use feature selection toolbox such as “tsfresh” or “hctsa” to systematically select the features [63,64]. A study on FMG feature extraction is warranted.

### 3.2. Predict Limb Action Using Machine Learning Techniques

To generate a machine learning model to predict limb action, the supervised machine learning technique is often used. The supervised approach requires a training set, which is associated with known actions. Using the collected dataset, a machine learning algorithm is then used to generate a model, which minimizes the discrepancy between prediction results and the true value. There are two categories of machine learning algorithms, one called classification used to predict discrete states such as hand gestures, and another one called regression to predict continuous parameters, such as finger movements. In this section, we discuss the algorithms used for FMG applications based on these two categories.

#### 3.2.1. Classification

**Figure 10:**
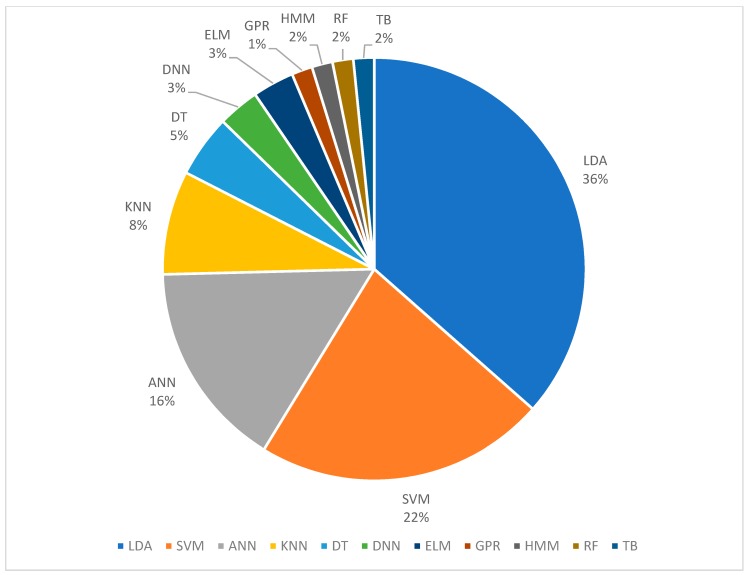
Percentages of the different classification algorithms reported in FMG literature (*n* = 63).

The percentages of different classification algorithms used in FMG literature is shown in Figure 10. The percentages shown in the figure include some of the work that used multiple classifications algorithms for performance comparison; the percentage is based on the total instance that each method was used and not on the number of publications. As shown in the figure, 37% of the publications use linear discriminant analysis (LDA), 23% use support vector machine (SVM), 15% use artificial neural network (ANN), and the rest use k-nearest neighbor (KNN) [9,31,37], decision tree (DT) [9], deep neural network (DNN) [59,60], extreme learning machine (ELM) [28,55], Gaussian process regression (GPR) [65], hidden Markov model (HMM) [9], random forest (RF) [33,66], and tree bagging (TB) [67].

LDA is a relatively straightforward and efficient learning algorithm that exists in the machine learning realm, making it suitable for implementation in low-power computation platforms for real-time control applications. Also, some FMG publications found that LDA had superior or comparable performance compared to the more complex algorithms. For example, Fang et al. reported that LDA had 5% higher accuracy than ANN for predicting six gestures using piezoelectric-based FMG sensors [58]. Ahmadizadeh et al. reported that LDA was comparable to SVM with no statistical difference when used for FMG-based prosthesis control [31]. Similar results were also obtained from one of our works for predicting wrist, forearm, and elbow positions [45] and from the work of Sadarangani et al., for detecting grasping in individuals with mild to moderate upper extremity impairment due to stroke [36]. When combining FMG signals with Leap motion controller for virtual grasp detection, LDA also had accuracy performance that was comparable to SVM, ANN, and tree bagger [67].

SVM is another efficient algorithm to classify input data once it is trained. For FMG applications, it was often reported to have superior performance when compared to the others [31,36,45,67]. However, in order to achieve high performance, some of the hyperparameters need to be fine-tuned using the cross-validation method, which is a computationally-intensive process.

ANN is the third most popular approach among FMG researchers. In general, it contains three layers, which are the input, hidden, and output layers. Each layer has a certain number of nodes that link between the adjacent layers. The associated weight for each node needs to be learned iteratively using back-propagation with gradient descent techniques. The iterative learning process allows for batch learning instead of feeding the entire learning data at once, making ANN highly adaptable. Furthermore, new data can be added to tune the model parameters at any time. However, no FMG related publications reported the use of such property. The configuration and the number of the hidden layers of ANN are highly configurable. When there multiple hidden layers, we often refer to such a type of network as the deep neural network (DNN). DNN is most suitable when there is a large amount of data to be trained, and it was used in FMG applications [59,60].

Many other classification algorithms have been used in FMG applications, each with its unique property. There is no consistent winner among these classifiers, thus researchers need to decide which one to use based on the applicational constraints and rely on expert intuition.

#### 3.2.2. Regression

**Figure 11:**
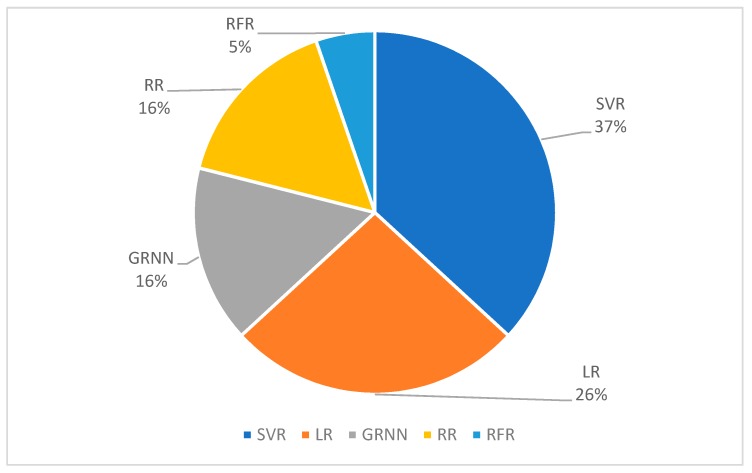
Percentages of the different regression algorithms reported in FMG literature (*n* = 19).

The percentages of different regression algorithms used in FMG literature is shown in Figure 11. Compared to classification, there is less variety in the regression algorithms found in the FMG literature. A total of 19 instances for using the regression method was found in 14 publications, 37% of them used support vector regression (SVR), 26% of them used linear regression (LR), 16% of them used ridge regression (RR), another 16% of them used general regression neural network (GRNN), and 5% of them use random forest regression (RFR).

SVR is the counterpart of SVM in the regression domain and both SVR and SVM are based on the same core principle, which is to identify the supported vector from the input data and form the model. SVR was used in the work of Castellini’s group to predict force exerted by the fingers [35,68], the work of Menon’s group to predict force exertion from the hand and dynamic finger movements [33,69,70], and in the work of Englehard’s group to predict upper limb movements with a high-density force-sensing matrix [48,71].

LR is a basic method to associate input signals and continuous output signals. The LR model is learned based on the least square method, which utilizes the pseudo-inverse technique. LR can be used as a filter to combine different input signals into one vector for further processing. For example, Curcie et al. used LR to filter FMG signals in order to distinguish different finger commands [6]. The same research group later used LR to predict grip force in the publication which first mentioned the term FMG [1]. Recently, the ability to predict grip force with LR using FMG signals was further studied by Stefanou et al. [72].

RR is an improved version of LR for regression applications. For RR, the input signals are first mapped into higher dimension features using an explicit transfer function or a kernel method. Then, the regression model is computed by using the least square method, which is the same as in the LR algorithm. RR has also been extensively used in FMG regression applications [35,40,68].

GRNN is the counterpart of ANN in the regression domain. It’s architecture and training method are almost identical to ANN, with the exception of the output layer, which does not have an activation function. GRNN was used in the work of Sakr et al., [40,69] and Kadkhodayan et al. [70].

RFR is a decision tree-based method for regression applications. Sadeghi et al. used it to predict dynamic finger movements, the results showing that RFR performs similarly than SVR, but requires much less time to train the model [33].

In order to improve regression performance for the targeted application, some researchers attempted to solve the problem with a two-step approach that utilized both the classification and regression methods. Specifically, Belyea et al. designed an experiment that allowed a participant to control the rotation and vertical movements of a virtual target based on wrist rotation and hand opening/closing actions, respectively [48,71]. The degrees of wrist rotation and hand opening/closing were predicted using FMG signals extracted from the forearm. For the one-step approach, the two parameters were regressed using SVR directly. In the two-step approach, the authors first used SVM to predict the intended action from one of the five possible movements, i.e., wrist pronation, wrist supination, hand open, hand closed, and no movement. Then, they selected a regression model to predict the degree of movement based on the predicted action. The results showed that such a two-step approach significantly outperformed the one-step approach. However, the two-step approach does not always guarantee better performance. For instance, Sadeghi et al. used FMG near the wrist to predict the dynamic movements of the index and middle finger when the wrist was in five different positions, i.e., neutral, extended, flexed, abducted and adducted [33]. The author used both the one-step and two-step approaches to predict the movements of the two fingers. In the one-step approach, the movement angles of the fingers were regressed directly and without consideration for different wrist positions. In the two-step approach, wrist position was first predicted by classifying the FMG signals. Based on the predicted results, a regression model was selected to predict finger movements. However, the authors found no statistically significant difference between the two approaches.

## 4. Discussion

In the past 20 years, researchers have investigated the capability of the FMG approach and developed methods to predict many gestures and limb movements. From studying the basic signal characteristics to the utilization of FMG for prosthesis control, progress has been made. However, there are still challenges that need to be overcome in order to use FMG technology in everyday scenarios. These challenges exist in all areas of FMG development, including both hardware and software, which are discussed in this section.

### 4.1. Challenges in FMG Hardware Development

The main challenge in FMG hardware development is to develop a device that can extract reliable FMG signals for a long period at a time. Two of the main factors affecting the reliability of the extracted signals are the sensor characteristics and the device configuration.

Currently, the majority of FMG research use sensors that are based on resistive polymer thick film (RPTF) technology to extract signals. This type of sensor is very compact in size and relatively inexpensive. However, such a sensor exhibits a strong non-linear characteristic against the actuation force and a large part-to-part error, making it difficult to directly link the sensor reading with muscle stiffness. Also, the majority of FMG devices are in the form of a strap or sleeve for user with intact limbs, which can be highly conformable to the user’s limb shape. However, because of its flexibility, it is difficult to ensure that sensors are donned exactly in the same location and with the same pressure each time. Furthermore, during long periods of operation, the sensors within the device may shift, worsening the reliability of the signals.

For limitations related to sensor properties, only an improvement in sensor fabrication technology can solve the problem. For research purposes, we can use high-quality sensors instead of the inexpensive RPTF sensors or develop our own custom solution. However, we also should keep in mind that one of the main advantages of using FMG to decipher limb activity is its low-cost factor. Therefore, if an engineer wants to utilize FMG technology to predict limb action, the tradeoff between sensor quality and cost may have to be considered.

Without careful calibration, it is difficult to control the preload force when the device is donned on a user. In most of published works, researchers adjusted the tightness of the device based only on the user’s oral feedback. The lack of objective assessment during the donning process introduces discrepancy in the FMG signal patterns between each setup routine. This limitation also contributes to the large variation in FMG patterns between different users. To reduce this discrepancy, we recommend extracting the averaged magnitudes of all FMG signals when the device was first donned, and then try to match this averaged value for subsequent donning routines. If the sensors used in the device were fully characterized and the output is converted into a standard pressure unit such as pascal, then a suitable range for the preload force can be identified for the targeted population. Using the standardized value, a feedback mechanism such as an LED indicator or a buzzer sound, can also be built onto the device to signal the right preload force.

To deal with the sensor shifting problem, researchers can improve on the design of the donning mechanism. However, they should also consider using smaller sensors that can be placed close to each other to form a map similar to the high-density FMG matrix presented by Radmand et al. [12] or Castellini et al. [14]. Such a matrix captures the full muscle stiffness characteristics of the targeted limb; any shifting of the device can be monitored by designing an algorithm to keep track of the shifting of a reference pattern. In this context, a reference pattern refers to a pattern associated with a known state that can be predicted with the highest confidence level. Once this state is detected, the current FMG signal pattern can be used to compare it with the one that was previously found. If the difference between two patterns is larger than a defined threshold, then the algorithm should adjust the machine learning model to account for the shifting. It is also important to note, the higher the spatial resolution the matrix has, the finer detail regarding the shifting of the sensor can be detected. However, with the higher spatial resolution and more sensing elements, more computational power is required. A study on identifying the optimum spatial resolution for the high-density matrix is warranted.

### 4.2. Challenges in FMG Software Development

The main use of FMG technology is to predict limb action for human-machine-interface or activity monitoring applications. Ultimately, we would like to use this approach to accurately predict as many actions as possible across all populations. Assuming we already have the best hardware to extract reliable FMG signals, we still need to process them to get reliable results. Besides the capability of the proposed machine learning algorithms, there are multiple factors influencing the ability to have accurate predictions. Two of the main factors are the intra-subject and the inter-subject variability of FMG signals.

The intra-subject variability refers to the change of FMG patterns due to the change of physical muscle condition during different activities. For example, after an exercise, our muscles may be fatigued, which changes the muscle stiffness pattern associated with the same action before the exercise. In order to combat this issue, we can use additional sensors such as skin temperature, sweat, movement, or sEMG sensors to assess the fatigue level. The history of the activity level can also be used as a predictor to assess fatigue. Once the fatigue level is assessed, we can then use this information to adjust the model parameters on the go. Such a scheme is within the realm of adaptive machine learning processes and it should be explored. That being said, a future study on how to assess the fatigue level for FMG applications is warranted.

The inter-subject variability refers to the difference in FMG patterns for a single action across different users. This difference is the result of a user’s physical condition across the whole population spectrum. For instance, individuals with an amputated limb or a limb that suffered from a severe physical injury or medical condition, their FMG signal patterns are expected to differ from an individual with a healthy intact limb. However, even within the group of individuals with a fully functional limb, FMG signal patterns can be quite different due to the difference in limb size, skin thickness, and muscle density, despite a similar anatomical configuration. These user-dependent characteristics have to be accounted for if we want to develop a general software algorithm for all users. The best solution is to capture large amounts of data from users with various physical conditions, across different age groups, and study the relations between these factors and FMG patterns. Researchers can also rely on deep learning techniques to develop a model that can be used for the general population. We believe that the deep learning approach has the potential to unleash the full capability of FMG technology to predict limb activity.

## 5. Conclusions

This review article has presented the state-of-art research and development of FMG technology in the past 20 years. 76-plus FMG related publications were found between 1999 and the middle of 2019. From the early exploration of the technique to its utilization in real-time control applications, much progress was made; yet, many challenges remain. We hope that this review article can provide new insight into FMG technology and contribute to its advancement.

## Figures and Tables

**Table 1:** Comparison chart between FSR and Flexiforce^®^ sensor.

	FSR (FSR402) [20]	Flexiforce^®^ (FLX-A201-F) [21]
Minimum actuation force (Newtons)	0.1	N/A
Force sensitivity range (Newtons)	0.1–10	0 to 4.4, 0 to 111, 0 to 445
Single part force repeatability	±2%	±2.5%
Part to part force repeatability	+/−6%	±40%
Hysteresis	+10%	<4.5%
Drift	<5% per log10 (time)	<5% per log10 (time)
Response time (micro seconds)	<3	<5
Linearity error	N/A	<±3%

## Appendix A

**Table A1:** List of FMG related publication.

Reference	Title	Publication Type
Abboudi et al., 1999 [3]	A biomimetic controller for a multi-finger prosthesis	Journal
Curcie et al., 2001 [6]	Biomimetic finger control by filtering of distributed forelimb pressures	Journal
Craelius et al., 2002 [5]	The bionic man: restoring mobility	Journal
Phillips et al., 2005 [4]	Residual kinetic imaging: a versatile interface for prosthetic control	Journal
Amft et al., 2006 [8]	Sensing Muscle Activities with Body-Worn Sensors	Proceeding
Lukowicz et al., 2006 [7]	Detecting and Interpreting Muscle Activity with Wearable Force Sensors	Proceeding
Ogris et al., 2007 [9]	Using FSR-based muscule activity monitoring to recognize manipulative arm gestures	Proceeding
Wininger et al., 2008 [1]	Pressure signature of forearm as predictor of grip force	Journal
Wang et al., 2010 [37]	Biomechatronic approach to a multi-fingered hand prosthesis	Proceeding
Yungher et al., 2011 [47]	Surface muscle pressure as a measure of active and passive behavior of muscles during gait	Journal
Li et al., 2012 [13]	Combined Use of FSR Sensor Array and SVM Classifier for Finger Motion Recognition Based on Pressure Distribution Map	Journal
Bin et al., 2012 [73]	Multi-sensor arm rehabilitation monitoring device	Proceeding
Morganti et al., 2012 [24]	A Smart Watch with Embedded Sensors to Recognize Objects, Grasps and Forearm Gestures	Proceeding
Yungher et al., 2012 [34]	Improving fine motor function after brain injury using gesture recognition biofeedback	Journal
Castellini et al., 2012 [74]	Intention Gathering from Muscle Residual Activity for the Severely Disabled	Proceeding
Dementyev et al., 2014 [30]	WristFlex	Proceeding
Radmand et al., 2014 [12]	High-resolution muscle pressure mapping for upper-limb prosthetic control	Proceeding
Castellini et al., 2013 [75]	Using a high spatial resolution tactile sensor for intention detection	Proceeding
Xiao et al., 2014 [28]	Towards the development of a wearable feedback system for monitoring the activities of the upper-extremities	Journal
Carbonaro et al., 2014 [76]	An Innovative Multisensor Controlled Prosthetic Hand	Proceeding
Castellini et al., 2014 [10]	Proceedings of the first workshop on Peripheral Machine Interfaces: going beyond traditional surface electromyography	Journal
Castellini et al., 2014 [68]	A wearable low-cost device based upon Force-Sensing Resistors to detect single-finger forces	Proceeding
Ravindra et al., 2014 [35]	A Comparative Analysis of Three Non-Invasive Human-Machine Interfaces for the Disabled	Journal
Rasouli et al., 2015 [55]	Stable force-myographic control of a prosthetic hand using incremental learning	Proceeding
Sadarangani et al., 2015 [27]	A wearable sensor system for rehabilitation applications	Proceeding
Koiva et al., 2015 [77]	Shape conformable high spatial resolution tactile bracelet for detecting hand and wrist activity	Proceeding
Sanford et al., 2015 [78]	Surface EMG and intra-socket force measurement to control a prosthetic device	Proceeding
Chengani et al., 2016 [79]	Pilot study on strategies in sensor placement for robust hand/wrist gesture classification based on movement related changes in forearm volume	Proceeding
Cho et al., 2016 [41]	Force Myography to Control Robotic Upper Extremity Prostheses: A Feasibility Study	Journal
Connan et al., 2016 [23]	Assessment of a Wearable Force- and Electromyography Device and Comparison of the Related Signals for Myocontrol	Journal
Jiang et al., 2016 [50]	Ankle positions classification using force myography: An exploratory investigation	Proceeding
Jiang et al., 2016 [32]	Exploration of Force Myography and surface Electro Myography in Hand Gesture classification	Journal
Li et al., 2016 [56]	FMG-based body motion registration using piezoelectric sensors	Proceeding
Radmand et al., 2016 [46]	High-density force myography: A possible alternative for upper-limb prosthetic control	Journal
Yap et al., 2016 [38]	Design of a wearable FMG sensing system for user intent detection during hand rehabilitation with a soft robotic glove	Proceeding
Kadkhodayan et al., 2016 [70]	Continuous Prediction of Finger Movements Using Force Myography	Journal
Sakr et al., 2016 [69]	On the estimation of isometric wrist/forearm torque about three axes using Force Myography	Proceeding
Ahmadizadeh et al., 2017 [31]	Toward Intuitive Prosthetic Control: Solving Common Issues Using Force Myography, Surface Electromyography, and Pattern Recognition in a Pilot Case Study	Journal
Ferigo et al., 2017 [42]	A Case Study of a Force-myography Controlled Bionic Hand Mitigating Limb Position Effect	Journal
Ghataurah et al., 2017 [43]	A Multi-sensor Approach for Biomimetic Control of a Robotic Prosthetic Hand	Proceeding
Jaquier et al., 2017 [65]	Combining Electromyography and Tactile Myography to Improve Hand and Wrist Activity Detection in Prostheses	Journal
Nowak et al., 2017 [66]	Multi-modal myocontrol: Testing combined force- and electromyography	Proceeding
Sadarangani et al., 2017 [36]	Force Myography for Monitoring Grasping in Individuals with Stroke with Mild to Moderate Upper-Extremity Impairments: A Preliminary Investigation in a Controlled Environment	Journal
Sanford et al., 2017 [80]	Concurrent surface electromyography and force myography classification during times of prosthetic socket shift and user fatigue	Journal
Xiao et al., 2017 [45]	Performance of Forearm FMG and sEMG for Estimating Elbow, Forearm and Wrist Positions	Journal
Xiao et al., 2017 [29]	Counting Grasping Action Using Force Myography: An Exploratory Study with Healthy Individuals	Journal
Delva et al., 2017 [81]	FSR based Force Myography (FMG) Stability Throughout Non-Stationary Upper Extremity Tasks	Proceeding
Booth et al., 2018 [82]	A Wrist-Worn Piezoelectric Sensor Array for Gesture Input	Journal
Castellini et al., 2018 [14]	Tactile Myography: An Off-Line Assessment of Able-Bodied Subjects and One Upper-Limb Amputee	Journal
Delva et al., 2018 [83]	Investigation into the Potential to Create a Force Myography-based Smart-home Controller for Aging Populations	Proceeding
Ha et al., 2018 [57]	Force Myography Signal-Based Hand Gesture Classification for the Implementation of Real-Time Control System to a Prosthetic Hand	Proceeding
Fang et al., 2018 [58]	Fabrication, structure characterization, and performance testing of piezoelectret-film sensors for recording body motion	Journal
Fujiwara et al., 2018 [60]	Optical fiber force myography sensor for identification of hand postures	Journal
Fujiwara et al., 2018 [84]	Optical fiber force myography sensor for applications in prosthetic hand control	Proceeding
Godiyal et al., 2018 [26]	Force Myography Based Novel Strategy for Locomotion classification	Journal
Jiang et al., 2018 [85]	Force Exertion Affects Grasp classification Using Force Myography	Journal
Jiang et al., 2018 [51]	Exploration of Gait Parameters Affecting the Accuracy of Force Myography-Based Gait Phase Detection	Proceeding
Jiang et al., 2018 [67]	Virtual grasps recognition using fusion of Leap Motion and force myography	Journal
Truong et al., 2018 [59]	CapBand	Proceeding
Zhang et al., 2018 [86]	A Pilot Study on Using Forcemyography to Record Upper-limb Movements for Human-machine Interactive Control	Proceeding
Belyea et al., 2018 [48]	A Proportional Control Scheme for High Density Force Myography	Journal
Sadeghi et al., 2018 [33]	Regressing grasping using force myography: An exploratory study	Journal
Anvaripour et al., 2018 [62]	Hand gesture recognition using force myography of the forearm activities and optimized features	Proceeding
Godiyal et al., 2018 [49]	A force myography-based system for gait event detection in overground and ramp walking	Journal
Belbasis et al., 2018 [48]	Muscle performance investigated with a novel smart compression garment based on pressure sensor force myography and its validation against EMG	Journal
Esposito et al., 2018 [22]	A piezoresistive sensor to measure muscle contraction and mechanomyography	Journal
Sakr et al., 2018 [40]	Exploratory Evaluation of the Force Myography (FMG) Signals Usage for Admittance Control of a Linear Actuator	Proceeding
Stefanou et al., 2018 [72]	Wearable Tactile Sensor Brace for Motion Intent Recognition in Upper-Limb Rehabilitation	Proceeding
Ha et al., 2019 [87]	Performance of Forearm FMG for Estimating Hand Gestures and Prosthetic Hand Control	Journal
Xiao et al., 2019 [61]	Does force myography recorded at the wrist correlate to resistance load levels during bicep curls?	Journal
Xiao et al., 2019 [25]	An Investigation on the Sampling Frequency of the Upper-Limb Force Myographic Signals	Journal
Xiao et al., 2019 [39]	Towards an FMG based augmented musical instrument interface	Proceeding
Belyea et al., 2019 [71]	FMG vs EMG: A Comparison of Usability for Real-time Pattern Recognition Based Control	Journal
Anvaripour et al., 2019 [88]	Controlling robot gripper force by transferring human forearm stiffness using force myography	Proceeding
Herrera-Luna et al., 2019 [11]	Sensor Fusion Used in Applications for Hand Rehabilitation: a Systematic Review	Journal
Prakash et al., 2019 [44]	Novel force myography sensor to measure muscle contractions for controlling hand prostheses	Journal
